# Influence of surface treatment on repair bond strength of CAD/CAM long-term provisional restorative materials: an in vitro study

**DOI:** 10.1186/s12903-023-03021-y

**Published:** 2023-05-30

**Authors:** Tarek Ahmed Soliman, Ali Robaian, Youssef Al-Gerny, Eman Mohamed Raffat Hussein

**Affiliations:** 1 Dental Biomaterials Department, Faculty of Dentistry, Mansoura University, Mansoura, Egypt; 2 Prosthetic Dentistry Department, Faculty of Dentistry, New Mansoura University, Mansoura, Egypt; 3 Conservative Dental Science Department, Faculty of Dentistry, Prince Sattam bin Abdulaziz University, Al-Kharj, Saudi Arabia; 4 Restorative Dental Science Department, Faculty of Dentistry, King Khalid University, Abha, KSA Saudi Arabia; 5 Prosthetic Dentistry Department, General Zagazig Hospital, El Sharkeya, Egypt; 6 Prosthetic Dentistry Department, Faculty of Dentistry, Misr International University, Obour, Greater Cairo, Egypt

**Keywords:** CAD-CAM, Provisional restoration, Shear Bond Strength, Surface conditioning

## Abstract

**Background:**

The surface treatment to improve the repair bond strength may vary because CAD/CAM provisional restoration polymers exhibit a variety of microstructures. This study was conducted to evaluate the effect of surface treatments on the repairability of three different CAD/CAM polymers for long-term provisional restorations.

**Methods:**

Thirty specimens from each provisional restorative materials (CAD-Temp, Everest C-Temp, and PEEK) were divided into three groups: C: surfaces received no treatment; SB: surfaces were airborne particle abraded with 50 μm aluminum oxide; SB-T: surfaces received the same conditions as group SB in addition to thermocycling before and after treatment. Primer and nanohybrid repair resin composite were applied to the prepared CAD /CAM surfaces. The shear bond strength and the mode of failure were assessed. ANOVA and Tukey’s significant difference tests were used to evaluate the data.

**Results:**

The SB group had significantly higher repair SBS values (p < .001) compared to the other groups (C and SB-T). Everest C-Temp significantly recorded the highest repair SBS (17.84 ± 0.19 MPa) in group SB, while the lowest repair SBS values (5.51 ± 1.14 MPa) for CAD-Temp were recorded in group C. PEEK significantly recorded the second highest repair SBS (15.96 ± 0.18) in the SB group.

**Conclusions:**

Everest C-Temp had the highest repair SBS after an airborne abrasion particle. Thermocycling had no significant effect on the repair SBS for PEEK. Everest C-Temp and PEEK are recommended as long-term durable provisional materials for clinical use.

## Introduction

There are many prefabricated polymer-based CAD/CAM materials in the market that can be used to fabricate provisional restorations. To improve material properties beyond conventional polymerization, prefabricated polymer blanks are industrially polymerized under standardized conditions at high temperature and pressure. Due to the increased fracture strength, better stress distribution, and less abrasion of the opposing enamel, these materials are more versatile [1–3].

Long-term provisional prostheses may present new therapeutic possibilities for maxillofacial rehabilitation, implant-supported treatments, and periodontal therapy [4–6]. Clinicians should consider the type of materials [7–9], simplicity of processing and bonding [10–12], mechanical properties, and oral environmental conditions [13] when choosing long-term provisional restorative materials. Although these restorations seem highly promising from a mechanical point of view, a resin composite veneering material is required for aesthetic reasons and their bonding durability in oral environments [14–16]. The durability of these CAD /CAM restorative polymers can be compromised by technical complications such as chipping, bond failure, and wear. These complications may lead to clinical failures and the need for additional operative treatment [12, 14].

When a fractured restoration is veneered with resin composite, the size and nature of the defect must be considered when the dentist decides whether to repair or replace the restoration [16]. The concept of repair is part of the philosophy of minimally invasive dentistry, which is based on the preservation of tooth structure and aims to avoid repeated cycles of restoration [17].

The repair requires special surface treatment because the interfacial adhesion between the new resin layers and the restoration degrades over time [18, 19]. Airborne-particle abrasion is a type of surface treatment to increase the surface area and wettability of the material, resulting in intimate adaptation of the resin/restoration interface and increased the repair bond strength. Because CAD/CAM interim restoration polymers demonstrate varied microstructures, the response to different surface treatments to enhance their repair bond strength may also vary [11, 12, 14]. Furthermore, there is scarce information available on the repairability of long-term provisional CAD/CAM restorations. As a result, the current study investigated the effect of surface treatments on the repairability of three different CAD /CAM polymers for long-term provisional restorations. The null hypotheses tested were that (1) there would be no significant difference among repair bond strength of the three different CAD/CAM materials, (2) the airborne particle abrasion does not affect repair bond strength, and (3) thermocycling does not affect repair bond strength.

## Materials and methods

In this study, three types of CAD/CAM polymers for long term interim restorations were used; Polyacrylate polymer (CAD-Temp; CT), fiber-glass-reinforced polymer (Everest C-Temp; ET), and Polyetherether ketone (PEEK, BioHPP; PK) (Table 1). A sample size of ten specimens in each group was necessary to provide a 0.95 power using a 0.05 threshold of significance, according to the power analysis (size effect = 2.34, -two tailed=. 05) [12].

**Table 1:** Materials used in the study

Product	Composition/ Manufacturer	Indication	Lot. No.
CAD-Temp	-83–86 wt.% PMMA, 14 wt.% micro filler (silica), Pigments (< 0.1%). - VITA Zahnfabrik.	Multi-unit, fully or partially anatomical long-term temporary bridges with up to 2 pontics.	38590
Everest C-Temp	-Fiber glass-reinforced polymer. - High performance endless molecular Polymer chain plastic. - KaVo, Biberach, Germany.	Long-term temporary restoration up to 6 units.	6946
PEEK (Bre CAM Bio HPP)	-Poly ether ether ketone, 20wt% titanium dioxide ceramic filler and Aluminum oxide sand (50 µm mean particle size) - Bredent GmbH &co., senden, Germany.	4-part posterior bridge up to two pontics.	56654456
Visio. Link Primer	-MMA, PETIA, photoinitiators -Bredent GmbH & Co., Senden, Germany.	Preparation of the adhesive bonds of high-performance polymers and PMMA materials.	153141
Filtek Supreme XTE	-Matrix: Bis-GMA, UDMA, TEGDMA, PEGDMA, Bis-EMA. -Filler load: 63.3 vol%, 78.5 wt% -3M ESPE, St. Paul, USA	Repair of acrylic and resin materials.	N470318

PMMA, poly methyl methacrylate; MMA: methyl methacrylate, PETIA; pentaerythritol—triacrylate; Bis-GMA, Bisphenol A glycidyl dimethacrylate; UDMA, urethane dimethacrylate; TEGDMA, triethyleneglycol dimethacrylate; PEGDMA, poly(ethylene glycol) dimethacrylate; Bis-EMA, Bisphenol A polyethylene glycol diether dimethacrylate

### Specimen preparation and grouping

For the experimental setup, thirty specimens (10 × 3 mm) of each type of provisional CAD/CAM material were produced using an ISOMET (Techcut4, Allied, USA). To ensure uniform specimen thickness, a digital caliper (Mitutoyo Corporation, Tokyo, Japan) was used. After finishing the bonded surfaces of the specimens using silicon carbide papers in grit levels ranging from 600 to 2000, distilled water was used for a 3-minute ultrasonic cleaning procedure. One side of the specimens was left exposed for surface treatment and bonding procedures, and then they were fixed in acrylic resin blocks (Paladur, Heraeus-Kulzer, Hanau, Germany) [3, 6].The experimental specimens were divided into one of the following three subgroups (n = 10) (Table 2) based on surface pretreatments: C; Surfaces of the specimens were not subjected to any surface treatment, SB; Surfaces were airborne particle abraded with 50 μm aluminum oxide (LEMAT NT4, Wassermann, Germany) for 10 s at a distance of 10 mm with a pressure of 0.55 MPa, and then air-dried for 20 s [14, 20, 21], and SB-T; Surfaces of the specimens were subjected to the same procedures as group SB with the addition of thermocycling before and after pretreatment. 5000 thermocycles (SD Mechatronic GmbH, Feldkirchen Westerham, Germany) at temperatures ranging from 5 to 55 °C for dwell times of 30 s and transfer times of 10 s were applied to group SB-T before and after repairing [14].

**Table 2:** Experimental groups in the study

Groups	Substrate	Repair steps				
		Thermocycling (TC) step before repair	Surface Treatment	Primer application	Repair step	Thermocycling (TC) step after repair
**C**	• CAD-Temp • Everest C-Temp • PEEK	---	---	✓	✓	---
**SB**	• CAD-Temp • Everest C-Temp • PEEK	---	✓	✓	✓	---
**SB-T**	• CAD-Temp • Everest C-Temp • PEEK	✓	✓	✓	✓	✓

C; Control groups (no pretreatment and no aging), SB; 50 µm airborne abrasion and primer, SB-T: the same condition as group SB in addition to thermocycling before and after pretreatment

### Bonding procedure

According to the manufacturer’s recommendation, Visio. Link primer (Bredent GmbH & Co., Senden, Germany) was applied with a micro brush and then immediately polymerized for 20 s using a LED light (Elipar Freeligh 2, 3 M ESPE, 1,226 mW/cm²). Using a 6-mm diameter circular split Teflon mold, a nanohybrid repair resin composite (Filtek Supreme XTE) was packed onto treated CAD/CAM surfaces. To fill the mold, 2-mm thick resin composite layers were incrementally applied, and each layer was light-cured. Following that, the SB-T specimens were subjected to 5000 thermocycles between 5 and 55 °C for a dwell time of 30 s and a transfer time of 10 s [14].

### Shear bond strength (SBS) test

**Fig. 1:**
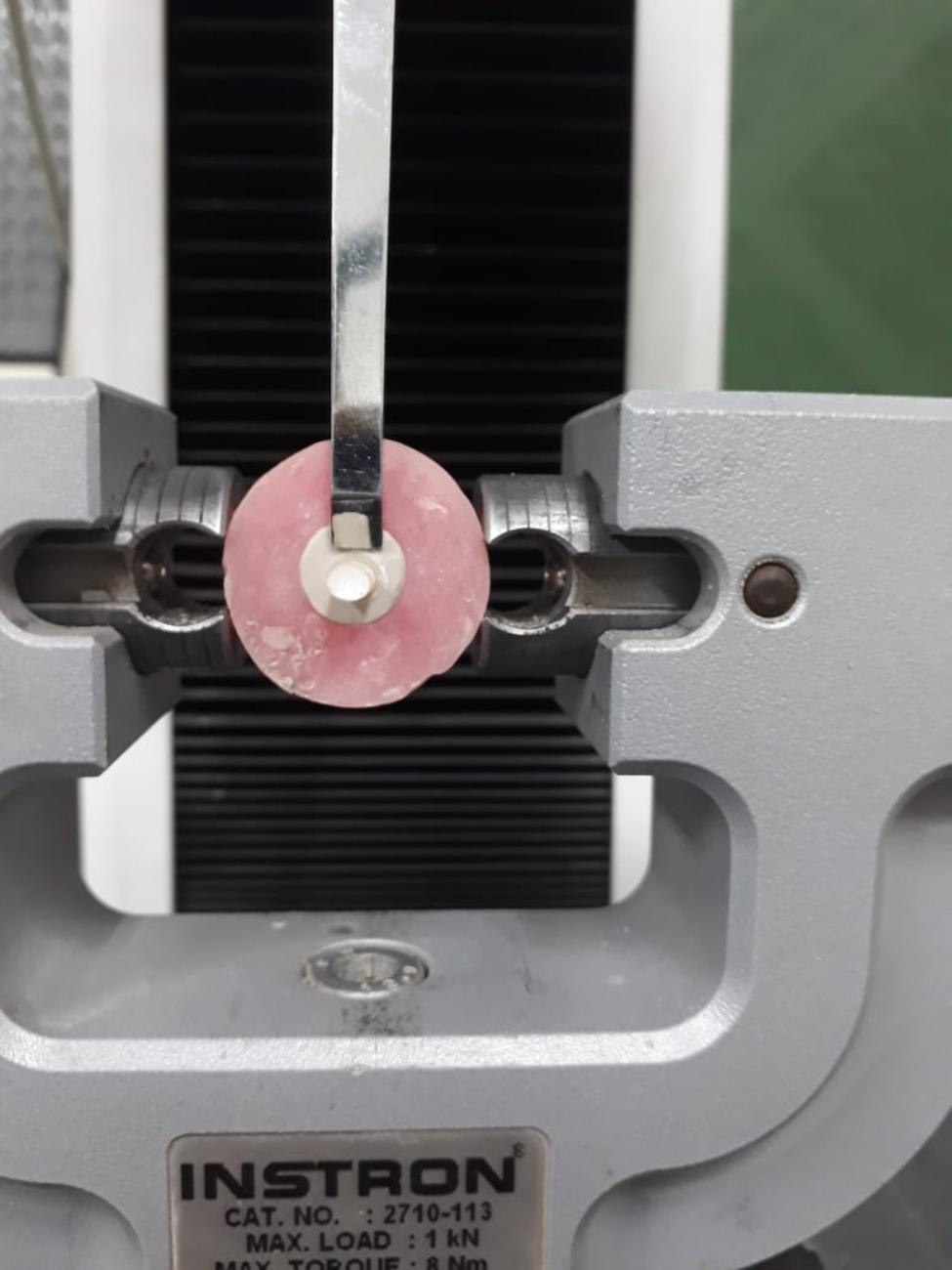
Shear Bond Strength Testing

The SBS test was carried out on a universal testing machine (AGS-1000 A; Shimadzu CO., Kyoto, Japan). The bonded CAD/CAM polymer-resin composite assembly was positioned in the machine’s lower jaw so that it was parallel to the direction of the shear force (Fig. 1). At a crosshead speed of 0.5 mm/min, a compressive loading was applied. The testing device’s upper moveable compartment was attached to a stainless-steel rod with a mono-beveled chisel configuration, and this rod was precisely positioned on the interface [3, 22, 23]. The testing machine displayed in Newton (N) the shear force at fracture (the force level at which the specimen debonds) using a 2.5 kN load cell connected to a computer. By dividing the fracture load (F) in Newton by the bonded surface area (A) in mm^2^, the SBS in megapascals (MPa) was computed. The bonded surface area was computed using a digital caliber (Mitutoyo Corporation, Tokyo, Japan). After debonding, the mode of failure was determined by examining the fractured specimen under a 20-x optical stereomicroscope (Olympus SZ61, Tokyo, Japan) and was divided into three categories: cohesive failure within the resin composite, adhesive type failure at the interface, and mixed type failure.

### Scanning electron microscopy evaluation

Three additional representative specimens from each material (10 mm × 10 mm × 1 mm) were prepared and cleaned with 96% ethanol in an ultrasonic bath for two minutes, then air-dried to assess the surface topography both before and after airborne particle abrasion. For the qualitative investigation of each material, specimens were mounted on metallic stubs, gold sputter-coated, and then examined under a SEM (Jeol-JSM-6510, Tokyo, Japan) with an initial magnification of 1000 x [3, 24].

### Statistical analysis

The normality and equal variance assumptions were fulfilled according to the Shapiro–Wilk test and Levene’s test. Subsequently, statistical analyses (SPSS 22.0; IBM statistics) of the shear bond strength were analyzed using Two-way ANOVA to determine statistically significant differences and to detect the interaction between the two independent variables (material type and the surface conditioning). Tukey’s significant difference test was used for *post-hoc* comparisons. The level of significance was set at 5% for all statistical tests.

## Results

The means and standard deviations of repair SBS values (MPa) for all groups are presented in Table 3. One-way ANOVA showed significant differences (*p* < .001) among the three-surface conditioning sub-groups for the three tested materials. Tukey’s multiple comparison tests showed that, the highest mean repair SBS values (17.84 MPa) were recorded for Everest C-temp in group SB, whereas the lowest repair SBS values (5.51 MPa) were recorded for CAD-temp in group C. SB group recorded significantly higher repair SBS values (*p < .001*) compared to other groups (C and SB-T) for CAD-temp and Everest C-temp. No significant differences (*p*$>$*0.05)* were detected between SB and SB-T groups for PEEK material. For CAD-temp, the highest mean repair SBS values were recorded for group SB (10.96), whereas the lowest repair SBS values were recorded for group C (5.551). For Everest C-temp, the highest mean repair SBS values were recorded for group SB (17.84), whereas the lowest repair SBS values were recorded for group SB-T (9.88). As for PEEK, the highest mean repair SBS values were recorded for group.

**Table 3:** Means (standard deviations) of Repair Bond Strength of Experimental Groups

Groups	Materials		
	CAD-Temp	Everest C-Temp	PEEK
C	5.51 ^cC^ (± 1.14)	11.92 ^bA^ (± 0.18)	8.67 ^bB^ (± 1.08)
SB	10.96 ^aC^ (± 0.19)	17.84 ^aA^ (± 0.19)	15.96 ^aB^ (± 0.18)
SB-T	7.94 ^bC^ (± 0.99)	9.88 ^cB^ (± 0.18)	17.19 ^aA^ (± 1.12)

Mean values represented with different superscript lowercase letters (column)

for each type of group is significantly different according to Tukey test (*P < .05)*

Mean values represented with different superscript uppercase letters (row)

for each type of material is significantly different according to Tukey test (*P < .05)*

SB-T (17.84), whereas the lowest repair SBS values were recorded for group C (8.67). Independent variables (Material type and surface conditioning) and their interactions were significantly affecting repair SBS values as shown by the two-way ANOVA table (Table 4).

**Table 4:** Two-way ANOVA table for repair shear bond strength (MPa)

Source of variations	Sum of squares	*df*	Mean squares	*F*	*P value*
Type of material	667.61	2	333.81	610.46	*p < .001*
Surface pretreatment	587.15	2	293.58	536.89	*p < .001*
Type of material x Conditioning	220.97	4	55.24	101.03	*p < .001*
Total	14457.72 90				

The modes of failure frequencies in all groups and materials are presented in Table 5. Regarding the type of materials, the highest percentage of adhesive failures were observed in CAD-Temp, while the highest percentage of mixed and cohesive failures were observed in Everest C-Temp and PEEK, respectively. Regarding the surface treatment groups, adhesive failures were the most prominent type of failure (100%) in the control group at CAD-Temp. In the air-borne particle abrasion, mixed type of failure was the prominent type in Everest C-Temp (50%) and PEEK (60%). In the air-borne particle abrasion after thermocycling, adhesive failure was the predominant type in CAD-Temp (80%), while mixed types of failures occurred in Everest C-Temp (50%) and PEEK (70%).

**Table 5:** Failure pattern of the experimental groups

Groups	Materials	Failure %		
		Adhesive	Mixed	Cohesive
C	CAD-Temp	100	0	0
	Everest C-Temp	0	80	20
	PEEK	60	30	10
SB	CAD-Temp	60	20	20
	Everest C-Temp	10	50	40
	PEEK	20	60	20
SB-T	CAD-Temp	80	20	0
	Everest C-Temp	30	50	20
	PEEK	20	70	10

**Fig. 2:**
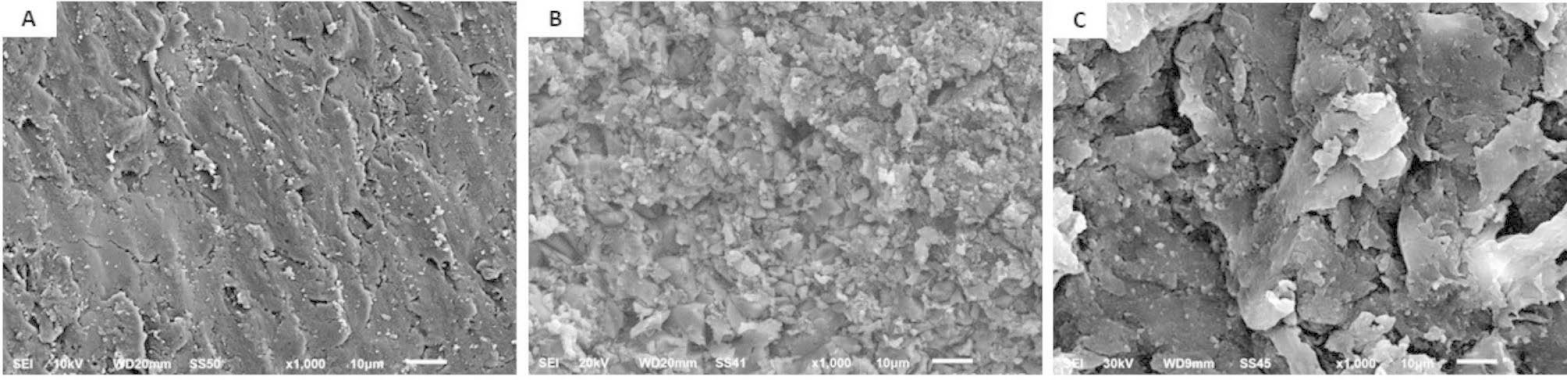
SEM micrographs (1000- X) of CAD-Temp **(A)** Everest C-Temp **(B)** and PEEK **(C)**

The surfaces of CAD-Temp, Everest C-temp, and PEEK under SEM showed variations in the surface microstructures (Fig. 2). CAD-Temp showed spherical areas of widely varying extensions that were obviously embedded in the resin matrix material (Fig. 2A). While Everest C-temp group showed irregular shaped long fibers intervening between them spherical shaped fillers particles with homogenous distribution within the materials (Fig. 2B). As for the PEEK group, it showed homogenous topography without voids or morphological defects (Fig. 2C).

## Discussion

In this study, three different CAD/CAM materials for long-term provisional restoration were investigated. These materials need to be layered with a resin composite and are recommended by their manufacturers as framework materials for implant-supported fixed prostheses. Repair restorations are typically required after months or years of clinical service since they were thermally cycled extensively in intraoral conditions. Thermocycling might reduce the residual monomer content by reducing the number of carbon-carbon double bonds. Moreover, thermocycling might lead to mechanical stress on the bonding area of the repaired substrate. However, it is also suggested that thermocycling may improve repair bond strength by increasing the post-polymerization process between polymeric CAD/CAM materials and adhesive resins [14, 25]. Additionally, there is insufficient information available regarding their repairability.

Depending on the reason and extent of the restoration fracture, intraoral repair with resin composite may be a simple and less expensive alternative to extraoral repair. Repair of restorations requires the surface to be pretreated to enhance the adhesion of repair resin composite to restorations [12, 14]. A variety of factors influence the bond strength of the repaired restoration, including the substrate’s surface condition and its chemical microstructure. The shear bond strength test is a dependable and simple in vitro test method for determining the bond strengths of materials with a relatively large surface area (typically 3–6 mm in diameter) [18]. As a result, shear bond strength tests were performed in this study to assess the bond strength of repaired specimens [26, 27].

Thermocycling is an in vitro simulation of the moist oral environment to test the durability of bonding prior to clinical recommendation [19, 28]. Therefore, the ageing of restorations should be considered and included in the repair plan [28]. In this study, the specimens were aged through 5000 thermal cycles to obtain aged substrate surfaces. In addition, the experimental specimens were thermally aged again (5000 thermal cycles) after repair to mimic clinical conditions and evaluate the long-term durability of the bond by simulating six months of clinical.

use [29].

Numerous methods have been conducted to improve the surface properties of provisional materials, such as air abrasion, laser treatment, sulfuric acid etching, and so on. Although 98% sulfuric acid etching produced the highest bond strength with PEEK material, it is not clinically viable because of its corrosive activity. In this study, micromechanical retention can be provided through airborne particle abrasion. It has been reported that it is the easiest way to improve the microroughness and increase the surface area of polymer-based dental materials for sufficient bonding [30, 31]. As a result, the current study aimed to assess the effect of airborne-particle abrasion as a surface treatment and ageing on the repairability of three different CAD/CAM long-term provisional restoration polymers.

Based on the ANOVA test, the two independent variables (material type and surface treatment) revealed a statistically significant effect (p < .001) on the repair SBS values. Consequently, the first two null hypotheses were rejected.

The minimum acceptable SBS value at the interface between resin-based materials and the substrate is 5 MPa, according to the specifications of ISO 10,477 [32]. On the other hand, Beher et al. [33] suggested that the clinically acceptable SBS value is 10 MPa. This clinical requirement was met in all groups except the C and SB -T groups in CAD -Temp, the SB -T group in C-Temp, and the C group in PEEK. Bond strength levels can vary widely depending on study design; hence, it is advisable to proceed with caution when applying laboratory bond strength results to clinical criteria.

According to the results of the study, Everest C-Temp had significantly the highest repair SBS (17.84 ± 0.19) in the SB group among the three tested materials (Table 3). This could be attributed to the irregular surface topography, as shown in Fig. 2B, which could facilitate the penetration of the adhesive resin and thus improve the interlock between the substrate and the repair materials. Moreover, C-Temp has a higher glass fiber content and is a high performance continuous molecular plastic polymer chain suitable for both adhesive resin and repair material penetration. These results are consistent with the Wiegand et al. study [18], where it was suggested that the higher SBS with C-Temp may be due to the ability of the adhesive to penetrate glass fiber-related surface irregularities and improve retention. Regarding PEEK, it recorded significantly the second highest repair SBS because air particle abrasion altered the surface morphology of PEEK and facilitated adhesive resin penetration into the substrate, improving micromechanical interlock and potentially increasing bond strength [30, 31, 34].

Thermal cycling is widely utilized to mimic the frequently changing temperatures in the oral environment. These thermal changes may lead to a reduction in bond strength. In the present study, thermocycling considerably decreased the repair SBS of CAD-Temp. This could be explained by its high polymeric content (83–86 wt% PMMA), which is susceptible to water penetration between polymer chains’ gaps and separating them from one another, causing water absorption and ultimately leading to resin matrix softening that has a detrimental effect on SBS [3].Thermocycling, on the other hand, decreased SBS of C-Temp because wet environments cause a glass fiber’s surface to corrode as a result water penetrating through the polymer matrix, lowering mechanical properties and, as a result, bond strength [3, 35]. Additionally, thermocycling increased the SBS of PEEK since thermocycling may improve repair bond strength by increasing the post-polymerization process between polymeric CAD/CAM materials and adhesive resins [25].

The third null hypothesis was partially accepted because there was no statistically significant effect of thermocycling on SBS in PEEK material. Since they contain a highly cross-linked polymer with 20% ceramic filler (with a grain size of 0.3 to 0.5 m) that can penetrate and seal the space between the PEEK polymer’s chains, reducing chain mobility and minimizing water penetration, this may be explained by their low water sorption ability value. PEEK has a water sorption value of (≤ 6,5 µg/mm³), CAD-Temp has a value of (≤ 40 µg/mm³), and Everest C-Temp has a value of (9,6 µg/mm3) [36, 37]. Neim et al. [38] observed that PEEK is not significantly affected by thermocycling in their analysis of the effects of 5000 thermocycling cycles on the physicomechanical properties of numerous CAD/CAM restorative materials. Additionally, Libermann et al. [15] evaluated how different ageing processes affected the water sorption of several CAD/CAM polymers. They came to the conclusion that storage media had no statistically significant impact on PEEK’s ability to absorb water.

Any change in the surface features of the tested material may affect the SBS values [7, 21]. In this study, a high percentage of adhesive failures occurred at CAD-Temp due to the insufficient bond strength of the repair resin on CAD-Temp [7, 19]. Furthermore, Everest C-Temp and PEEK were found to have a higher incidence of mixed failures, owing to uneven shear force distribution at the resin-restoration interface. The results are supported by a shift from adhesive to mixed failure when bond strength was increased. Clinical relevance for successful bonding on CAD/CAM surfaces can be achieved by surface roughening and the selection of a suitable adhesive system.

The present study has some limitations; Only one type of repair veneering resin and one adhesive system were used, so the results of this study cannot be generalized to other commercially available repair systems. In addition, different surface treatments such as etching solutions and the use of different adhesive systems, need to be further investigated. Furthermore, long-term clinical performance should also be evaluated in a clinical study with a controlled, standardized study design.

## Conclusions

Within the limitations of this in vitro study, it is concluded that the three tested CAD/CAM polymers can be adequately repaired after airborne-particle abrasion surface pretreatment. Everest C-temp recorded the highest repair SBS after airborne particle abrasion. Although, repair SBS was significantly reduced by thermocycling in both CAD-Temp and Everest C-Temp, PEEK was not significantly affected. PEEK and Everest C-Temp can be recommended for clinical use as long term, durable provisional materials.

## Data Availability

This article has all the data that were collected or analyzed during this study.
